# Supplementary material for: Superior probabilistic computing using operationally stable probabilistic-bit constructed by a manganite nanowire
Source: Natl Sci Rev. 2024 Sep 23;12(3):nwae338. doi: 10.1093/nsr/nwae338 (PMC11881701; doi:10.1093/nsr/nwae338)
Supplement: nwae338_Supplemental_File [file nwae338_supplemental_file.pdf]

Supplementary Materials for

Superior probabilistic computing using operationally stable probabilistic-bit constructed by manganite nanowire

Yadi Wang *et al*

*Corresponding authors. Email: wcyu@fudan.edu.cn; hangwenguo@fudan.edu.cn; shenj5494@fudan.edu.cn

This PDF file includes:

Supplementary Text

Figs. S1 to S10

Table S1

Supplementary Text

Theoretical estimation of correlation between C_1 and C_2 in a genetic network

Taking C_1 and C_2 in the family tree as an example, the random variable X is defined as the genetic similarity. When a random event occurs where C_1 and C_2 share the same allele for a particular gene (either dominant or recessive), X takes the value of 1; otherwise, X takes the value of -1. The expected value of X, denoted as $\overline{X}$, represents the correlation. The probability that C_1 and C_2 take the same dominance or recessiveness can be derived as

$$\begin{aligned} P(C_1 = C_2) &= P(C_1 = A, C_2 = A) + P(C_1 = a, C_2 = a) \\ &= \sum_{FM} P(C_1 = A, C_2 = A, F, M) + \sum_{FM} P(C_1 = a, C_2 = a, F, M) \\ &= \frac{3}{4} \end{aligned} \tag{S1}$$

A and a represent the dominant and recessive genes, respectively. Likewise, the probability that C_1 and C_2 are different can be derived as:

$$\begin{aligned} P(C_1 \neq C_2) &= P(C_1 = A, C_2 = a) + P(C_1 = a, C_2 = A) \\ &= \sum_{FM} P(C_1 = A, C_2 = a, F, M) + \sum_{FM} P(C_1 = a, C_2 = A, F, M) \\ &= \frac{1}{4} \end{aligned} \tag{S2}$$

Therefore, the theoretical correlation between C_1 and C_2 according to Bayesian theory can be calculated as

$$\begin{aligned} \text{Correlation}_{\text{Baye}} &= (1) * \frac{3}{4} + (-1) * \frac{1}{4} \\ &= \frac{1}{2} \end{aligned} \tag{S3}$$

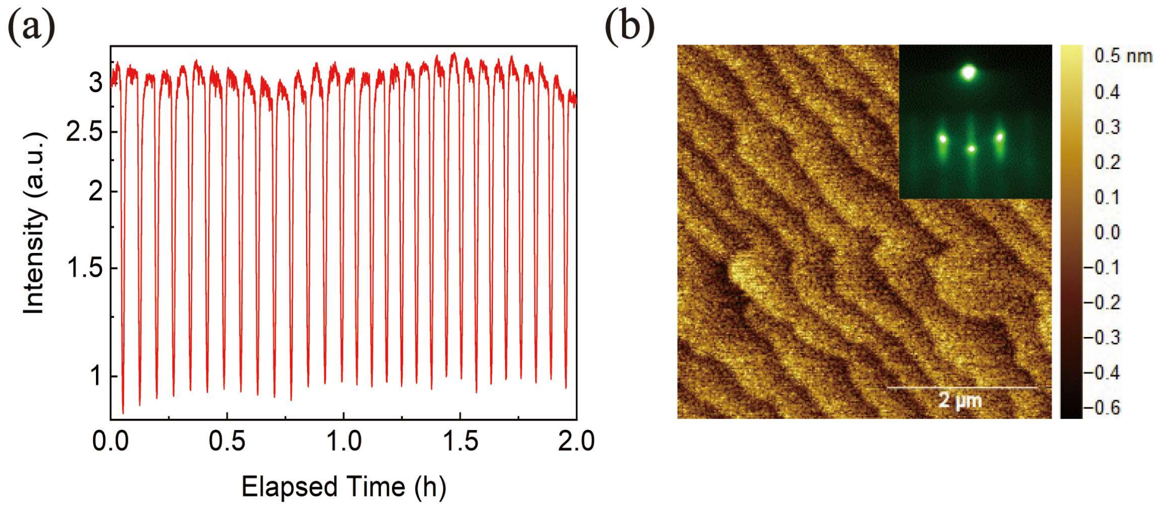

Fig. S1. Sample growth and characterization. (a) layer-by-layer growth of LPCMO thin film as monitored by reflective high energy electron diffraction (RHEED) on zero-order diffraction spot signal intensity. (b) atomic force microscopy (AFM) image of 40 nm LPCMO film, inset shows the RHEED pattern of film surface.

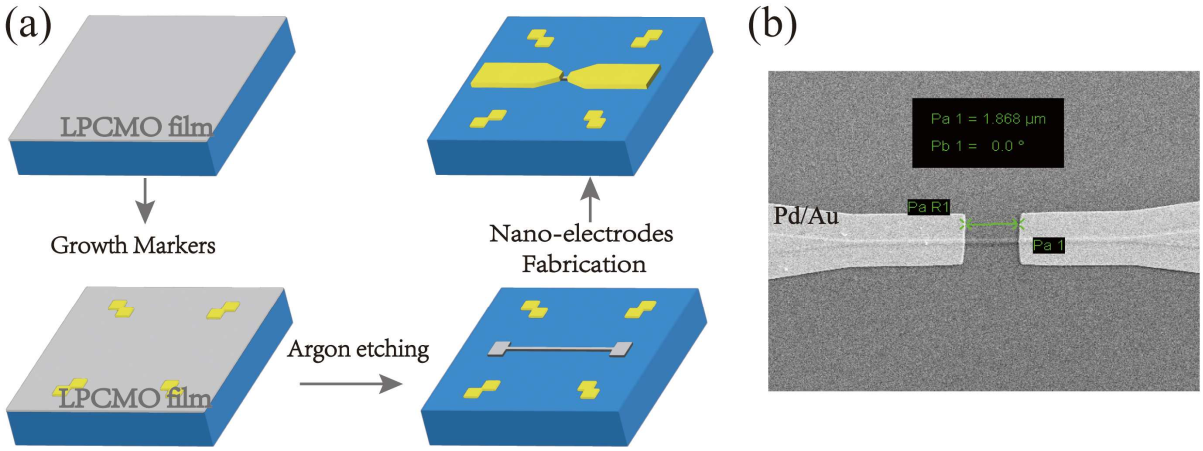

Fig. S2. Device fabrication and characterization. (a) schematics of device fabrication processes. (b) scanning electron microscopy (SEM) image of the nanowire device.

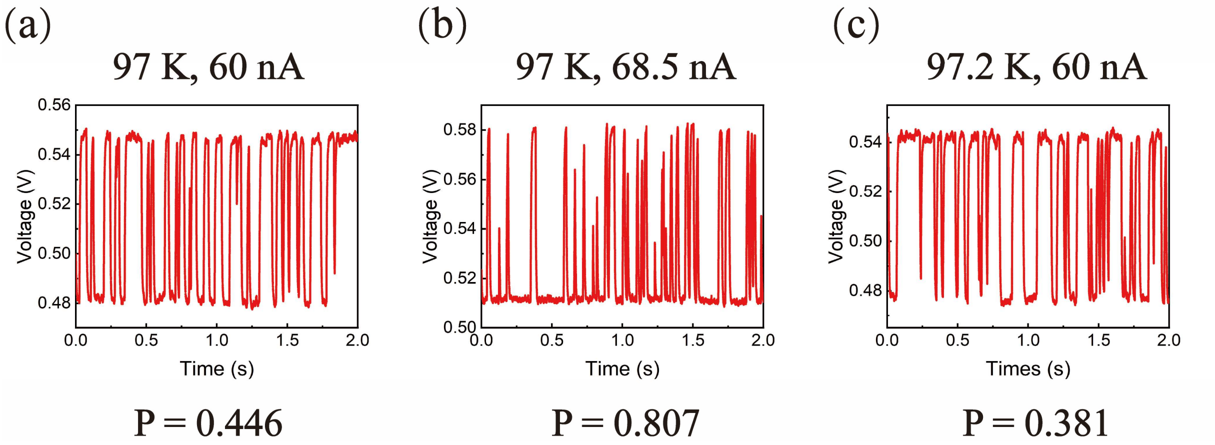

Fig. S3. Probability values at different temperatures and current.

Note: exclusion of Joule heating effects

Here, the effect of Joule heating on the current-tunable probability changes can be excluded. Figure S3a and b show the results for input current of 60 nA and 68.5 nA with probability values equal to 0.446 and 0.807, respectively. These results suggest the domain tends to switch more to the metallic phase with increasing input current, which is consistent with Figure 1d in the main text. If the above effect is caused by local Joule heating which rises the local temperature (for example, 0.2 K), then similar increment on the probability value will be observed at slightly higher temperature. In contrast, as shown in Figure S3c, when increasing the temperature to 97.2 K and keeping the input current at 60 nA, the probability decreases to 0.381. These results effectively rule out the effect of local Joule heating on our observation.

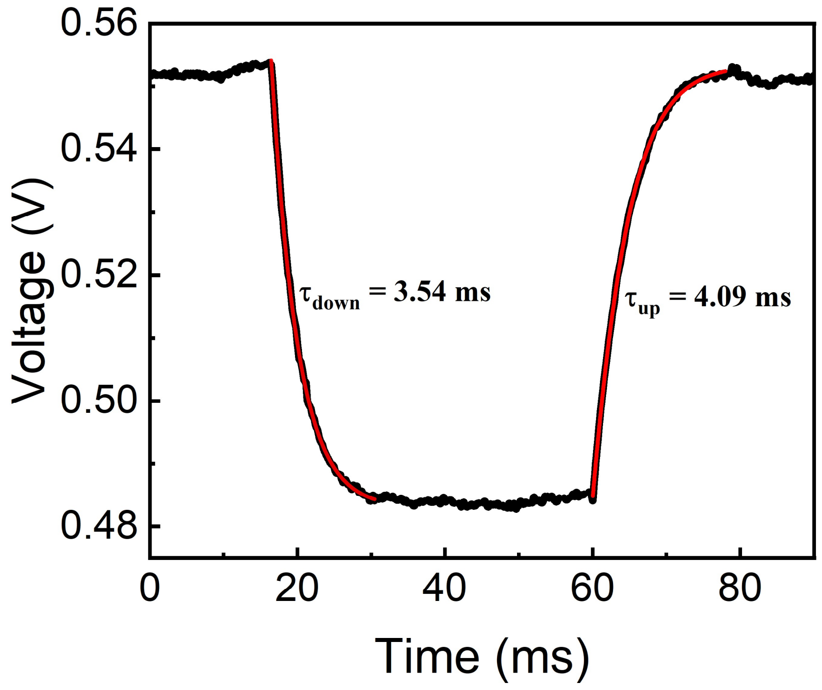

Fig. S4. Timescale of stochastic dynamics. The data are fitted with $V = A * e^{-t/\tau} + V_0$, where V is voltage, A and V_0 are constants, t is time, and τ is transition lifetime.

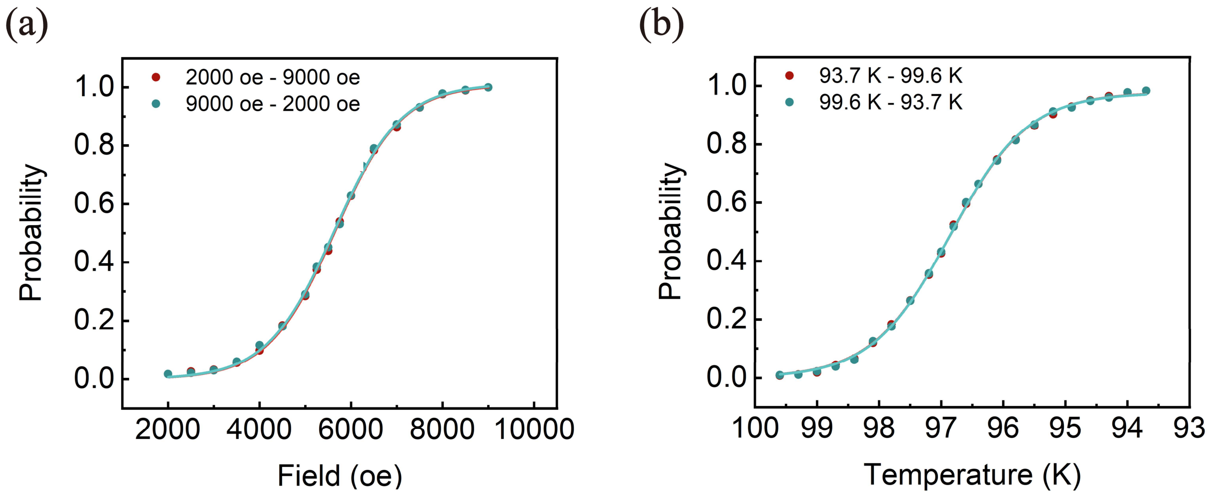

Fig. S5. Multi-field tunable p-bits. (a) Magnetic field and (b) temperature control of probability measured at output current of 40 nA. Red and green curves represent Sigmoid fits, respectively.

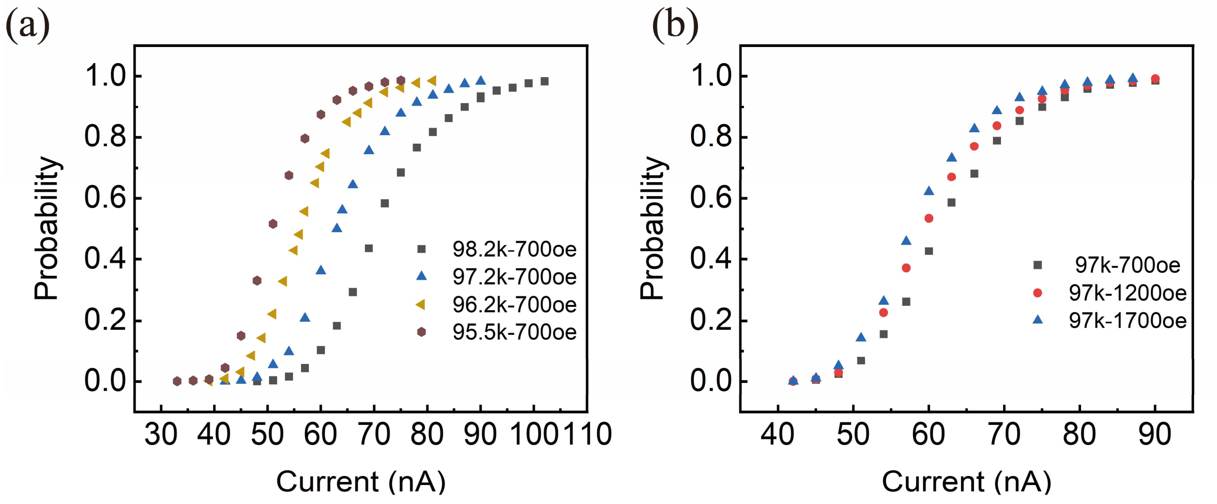

Fig. S6. Full range probability (0 to 1) as function of input current with different external conditions.

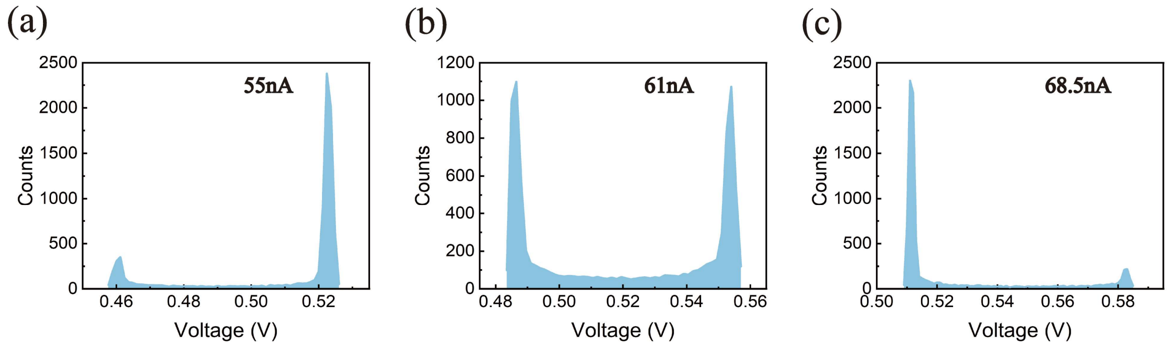

Fig. S7. Statistical diagram of voltage distribution for p = 0.208, 0.501, 0.807 respectively.

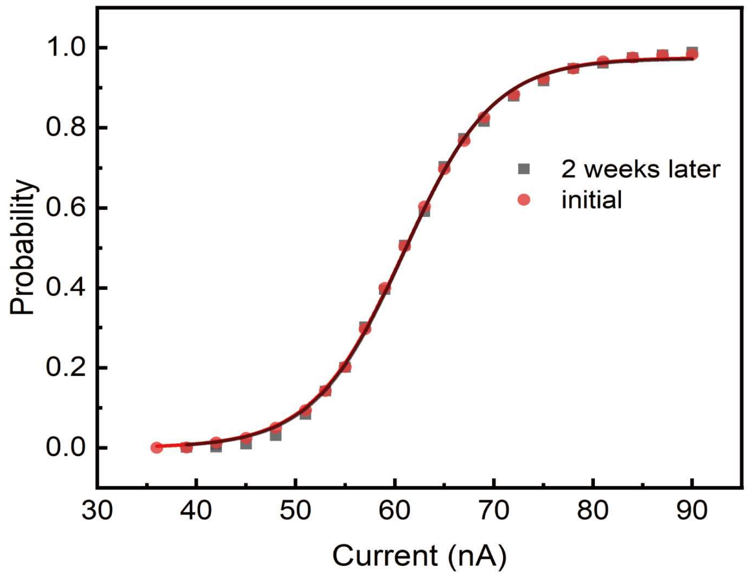

Fig. S8. Stability of p-bit after prolonged operations. The probabilistic curve and corresponding sigmoidal fit nearly coincides before and after 2 weeks of extensive operations.

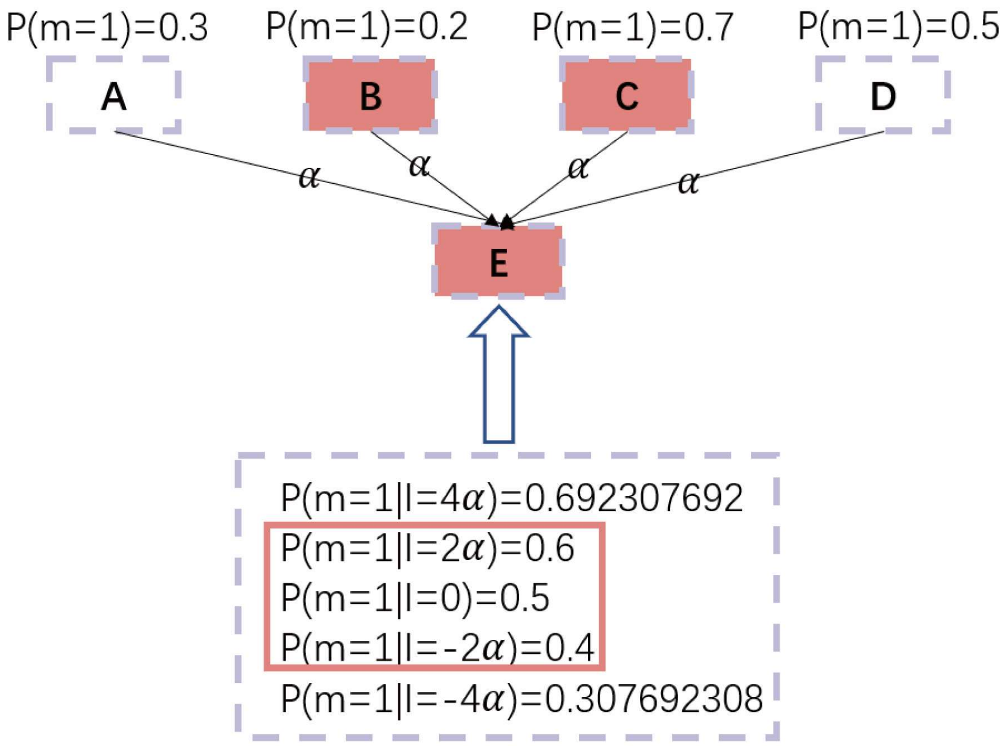

Fig. S9. Directed Graph Networks Comprising More Parent Nodes.

In the main text, we demonstrate the simplest case that each node is contributed by only two parent nodes, as depicted in the red section of Fig. S9, where node E has two parent nodes, B and C. Assuming the magnitude of all weights takes the same value denoted by α, the node E works in three different conditions that $I = 2\alpha, 0, -2\alpha$. We further investigate the case that node E has four parent nodes so that the number of working conditions is extended to five ($I = 4\alpha,\ 2\alpha, 0, -2\alpha, -4\alpha$), as shown in the dashed box of Fig. S9. The lower part of the figure illustrates the conditional probability of node E outputting 1 under these two network structures. Using the probability of node E outputting 1 at R=0, $M\rightarrow\infty$ as the theoretical value, we examine the relative error of the probability of node E outputting 1 for R=0, 0.0007, 0.05, 0.1 and M=1−50 in two networks. Subsequently, consistent with the main text, we calculated δ_{eff}. The results are shown in the Fig. S10.

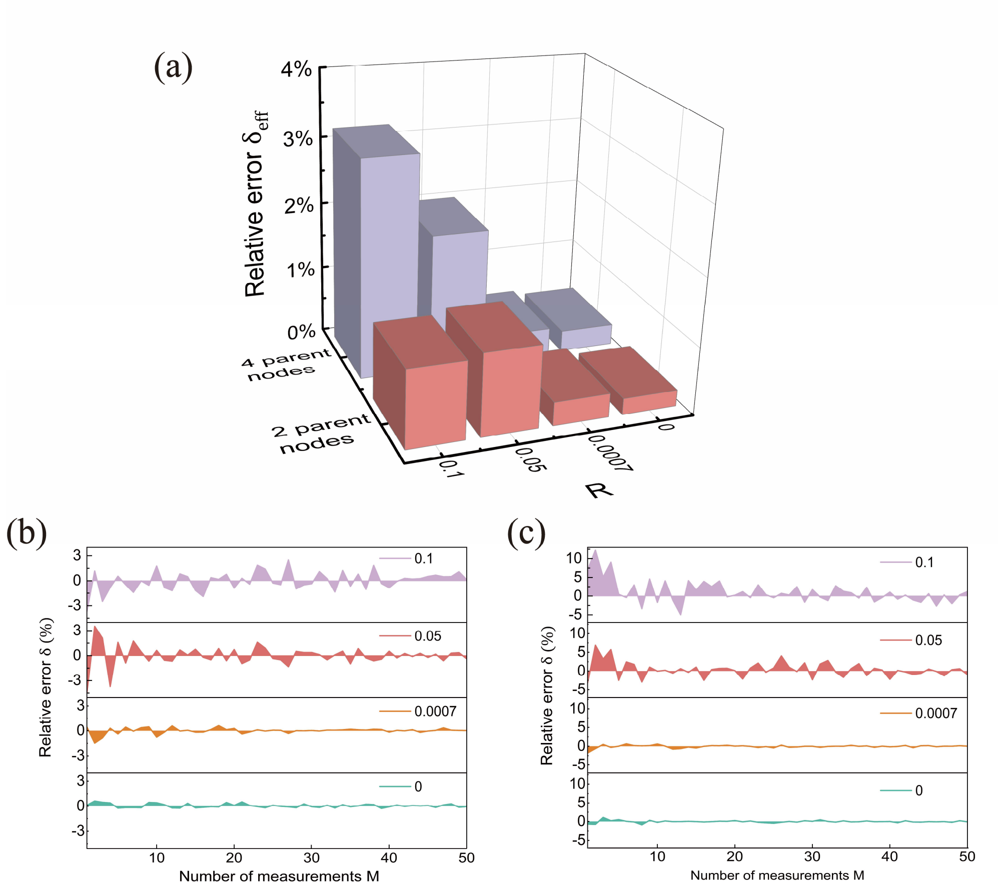

Fig. S10. (a) Performance of node E outputting 1 (relative error δ between numerical statistics and theoretical value) vs the number of nodes and broadening factor R (0, 0.0007, 0.05 and 0.1). Convergence of relative error δ for two parent nodes Network (b) and four parent nodes Network (c) with number of independent measurements M and different operational variation.

It can be observed from Figure S10 that as the number of parent nodes for node E as well as the value of R increases, the convergence of the results deteriorates. We analyze that this is because as the number of parent node variables increases, the number of terms required to compute the marginal probability $P(m_E = 1)$ increases, leading to cumulative errors. Therefore, for a realistic Bayesian tasks such as medical diagnosis where multiple parent nodes are usually considered, p-bits with high operational stability are of vital importance.

	PCM	MTJ	RRAM	LPCMO
maximum standard error	~10%[12]<br>~16%[3]	~14%[42]<br>~8%[43]	~19%[4]	1.3%
broadening factors *R*	~0.05<br>~0.1	0.05-0.1<br>~0.05	>0.1	0.0007

Table S1. The maximum standard error of sigmoid function and its corresponding broadening factors R of different types of p-bits.
